# A Cross-Sectional Study Demonstrating Increased Serum Amyloid A Related Inflammation in High-Density Lipoproteins from Subjects with Type 1 Diabetes Mellitus and How This Association Was Augmented by Poor Glycaemic Control

**DOI:** 10.1155/2015/351601

**Published:** 2015-10-18

**Authors:** Jane McEneny, Jane-Ann Daniels, Anne McGowan, Anjuli Gunness, Kevin Moore, Michael Stevenson, Ian S. Young, James Gibney

**Affiliations:** ^1^Centre for Public Health, Queen's University Belfast, Institute of Pathology, Grosvenor Road, Belfast BT12 6BJ, UK; ^2^Department of Endocrinology, Tallaght Hospital, Dublin 24, Ireland

## Abstract

Inflammatory atherosclerosis is increased in subjects with type 1 diabetes mellitus (T1DM). Normally high-density lipoproteins (HDL) protect against atherosclerosis; however, in the presence of serum amyloid-A- (SAA-) related inflammation this property may be reduced. Fasting blood was obtained from fifty subjects with T1DM, together with fifty age, gender and BMI matched control subjects. HDL was subfractionated into HDL_2_ and HDL_3_ by rapid ultracentrifugation. Serum-hsCRP and serum-, HDL_2_-, and HDL_3_-SAA were measured by ELISAs. Compared to control subjects, SAA was increased in T1DM subjects, nonsignificantly in serum (*P* = 0.088), and significantly in HDL_2_(*P* = 0.003) and HDL_3_(*P* = 0.005). When the T1DM group were separated according to mean HbA1c (8.34%), serum-SAA and HDL_3_-SAA levels were higher in the T1DM subjects with HbA1c ≥ 8.34%, compared to when HbA1c was <8.34% (*P* < 0.05). Furthermore, regression analysis illustrated, that for every 1%-unit increase in HbA1c, SAA increased by 20% and 23% in HDL_2_ and HDL_3_, respectively, independent of BMI. HsCRP did not differ between groups (*P* > 0.05). This cross-sectional study demonstrated increased SAA-related inflammation in subjects with T1DM that was augmented by poor glycaemic control. We suggest that SAA is a useful inflammatory biomarker in T1DM, which may contribute to their increased atherosclerosis risk.

## 1. Introduction

Patients with type 1 diabetes mellitus (T1DM) have an elevated risk of coronary atherosclerosis and coronary heart disease (CHD) that is not explained by conventional risk factors [1]. In contrast to patients with type 2 diabetes mellitus (T2DM), their typical lipid profile is normal or even apparently better than the general population, with increased high-density lipoprotein- (HDL-) cholesterol and decreased low-density lipoprotein- (LDL-) cholesterol and triglycerides [2]. However, these relatively simple lipid measurements potentially mask more subtle lipoprotein abnormalities, including disorders of lipoprotein function that might contribute to atherosclerosis in T1DM.

Serum amyloid-A (SAA) is an inflammatory protein that potentially contributes to dysfunctional HDL and progression of atherosclerosis. SAA has been detected in atherosclerotic lesions particularly in foam cells, is thought to be implicated in CHD [3] and may also indirectly cause plaque destabilization—an independent risk factor for cardiovascular disease (CVD) [4].

Studies investigating serum-SAA in subjects with T1DM have been inconclusive, some reporting increased levels [5] and others no difference [6]. However, to date, no studies have investigated if SAA is increased in HDL, specifically HDL_2_ and HDL_3_, in subjects with T1DM. There are several reasons for investigating SAA that is associated with HDL. Firstly, serum-SAA is reflective of both acute and chronic inflammation and, therefore, is influenced by short-term fluctuations in inflammation [7]. Secondly, SAA that is not associated with HDL is liable to proteolytic cleavage [8], which further influences its serum levels. Thirdly, as HDL has an approximate 4-day half-life in the circulation [9], SAA associated with this lipoprotein is more stable and thus more reflective of chronic low-grade inflammation. Finally and most importantly, because HDL function is impaired by SAA within the particle rather than in serum [10–12], direct measurement of HDL-associated SAA is necessary to demonstrate that it may be of pathological significance in T1DM.

Therefore, investigation of SAA in HDL subfractions in T1DM enhances our knowledge of its usefulness as a marker of inflammation and may also provide evidence of a mechanistic link between inflammation and atherosclerosis/CVD in these patients. To assess this, SAA was measured in serum, HDL_2_, and HDL_3_ in patients with T1DM and compared to well-matched control group. Further analysis was carried out to determine the contribution of glycaemic control on these variables.

## 2. Materials and Methods

### 2.1. Study Population

Patients with T1DM (*n* = 50) were recruited from the Diabetes Database in Tallaght Hospital, Dublin, Ireland, and were not reported to have preexisting CVD. Subjects without diabetes were recruited by local advertisement or were relatives of the T1DM patients. The inclusion criteria for subjects in the T1DM group were as follows: T1DM, between 20 and 45 years of age, and BMI less than 30 kg/m^2^, while the inclusion criteria for the control group were as follows: nondiabetic, between 20 and 45 years of age, and BMI less than 30 kg/m^2^. All subjects gave their written signed consent to the study, which was approved by the Research Ethics Committee of the Adelaide and Meath Hospital and St. James' Hospital (Dublin, Ireland).

### 2.2. Blood Processing

Blood was collected into standard serum tubes by the vacuette system and was allowed to sit at room temperature for a period of 30 mins to allow clotting. Serum was obtained by centrifugation at 3000 rpm for 15 mins at 4°C. The serum supernatant was removed and frozen in 1.3 mL aliquots in a −80°C freezer, until required for further analysis.

### 2.3. Primary Laboratory Analysis

Baseline measurements included fasting serum levels of glucose, total cholesterol, triglycerides, HDL cholesterol, and LDL cholesterol, which were measured using standard enzymatic assays on an automated ILab-600 biochemical analyser (Cobas Roche Diagnostics, West Sussex, UK). HbA1c was measured in serum by ion exchange HPLC and high-sensitivity C-reactive protein (hsCRP) was measured by an enzyme linked immunosorbent assay (ELISA) using a commercial available kit (BioCheck Inc., Foster City, USA). Height (cm) and weight (kg) were collected using a stadiometer and calibrated scales and used to determine BMI (kg/m^2^). These primary laboratory analyses were carried out in the laboratories of Tallaght Hospital, Dublin.

### 2.4. Isolation of HDL_2_ and HDL_3_ from Serum

HDL_2_ and HDL_3_ were harvested from freshly thawed serum by rapid ultracentrifugation, according to the method of McPherson et al. [13]. This is a 3-step, 6-hour long procedure.

### 2.5. Protein and Apo AI Determination

The protein concentration of HDL_2_ and HDL_3_ was determined spectrophotometrically using a commercial version of the Bradford assay (Bio-Rad, Hemel Hempstead, UK), as previously described [13]. Total protein concentration was utilised to standardise SAA within HDL_2_ and HDL_3_. Apo AI concentration was determined by single radial immunodiffusion, as previously described [13].

### 2.6. SAA Concentration in Serum, HDL_2_, and HDL_3_

SAA was measured in serum, HDL_2_ and HDL_3_ by a commercially available ELISA procedure (Invitrogen, KHA0011), which detects human-SAA1 and SAA2. The analysis was performed on a Grifols TRITURUS ELISA system (Italy), as per the manufacturer's instructions, with the following modifications. Serum was diluted 1 : 150, HDL_2_ 1 : 10 and HDL_3_ 1 : 100. The intra- and interassay precision of this assay were both <8%. This kit did not cross-react with a wide range of other proteins, including CRP, TNF-*α*, and IL-6.

### 2.7. Statistical Analysis

Statistical analyses were performed using SPSS (Statistical Package for the Social Sciences) Statistics version 21. Variables were assessed for normality and logarithmically transformed where required. Between-group analyses were analysed by independent* t*-test for normal (*n* = 49) versus T1DM subjects (*n* = 50) (described as comparison 1), and between T1DM subjects when separated according to HbA1c; that is, the T1DM group was separated into two groups according to mean HbA1c (<8.34% (68 mmol/mol) *n* = 24 or ≥8.34% (68 mmol/mol) *n* = 26). This secondary analysis is described as comparison 2. The relationship between HbA1c and SAA was further assessed by linear regression. Correlations were determined by Pearson's coefficient. All variables were summarised as mean (standard deviation, SD) when normally distributed and as geometric mean (interquartile range) when normally distributed after logarithmic transformation. Significance was set as *P* ≤ 0.05.

## 3. Results

### 3.1. Subject Characteristics

Subjects with hsCRP > 10 mg/L were removed from the analyses (*n* = 1 in control group) as this is suggestive of an active infection/inflammation and is in accordance with American Heart Association Guidelines [14].

Subject characteristics are shown in Table 1. These illustrate that when control subjects were compared to overall T1DM subjects (comparison 1), BMI, age, gender, total-cholesterol, and HDL-cholesterol were comparable between the groups (*P* > 0.05 for all analyses), while triglycerides and LDL-cholesterol were lower in T1DM (*P* ≤ 0.05 for both analyses). As was to be expected, fasting plasma glucose and HbA1c were higher in T1DM subjects (*P* ≤ 0.001 for both analyses). There were a small number of subjects in the T1DM group who were receiving statin therapy (5/50), while none of the control subjects were receiving lipid lowering therapy.

To explore the effect of glycaemic control in T1DM, we performed a further analysis where the T1DM group was subdivided into patients with HbA1c < (*n* = 24) or ≥ (*n* = 26) the median value (8.34%; 68 mmol/mol). The results for this analysis are presented as comparison 2 in Table 1, where BMI, age, gender, fasting glucose, triglycerides, HDL-cholesterol, and LDL-cholesterol were similar between the two T1DM groups (*P* > 0.05 for all analyses). However, total cholesterol was lower in the T1DM group with <8.34% HbA1C (*P* < 0.05). As anticipated HbA1c was lower in the T1DM subjects in the group with HbA1c < 8.34%, compared to the T1DM subjects with HbA1c ≥ 8.34% (*P* ≤ 0.001).

### 3.2. Total Protein and Apo AI Concentration in HDL_2_ and HDL_3_

The results illustrated that compared to control subjects, total protein was significantly higher in HDL_2_ (242 (90) versus 277 (86) mg/L; *P* = 0.047) and significantly lower in HDL_3_ (2720 (793) versus 2410 (614) mg/L; *P* = 0.033) in the overall T1DM group (comparison 1). Analysis in comparison 2 demonstrated that the difference in HDL_2_ protein resulted from higher levels in the T1DM group with HbA1c ≥ 8.34%, compared to T1DM group with HbA1c < 8.34% (295 (92) versus 259 (76) mg/L; *P* < 0.05), while the difference in HDL_3_ protein resulted from lower protein levels in T1DM group with HbA1c < 8.34%, compared to the T1DM group with HbA1c ≥ 8.34% (2271 (588) versus 2538 (620) mg/L; *P* < 0.05). HDL_2_ and HDL_3_ apo AI concentration were comparable between the control and T1DM groups in comparison 1 (HDL_2_, 162 (86) versus 146 (51) mg/L; *P* = 0.255: HDL_3_, 1590 (690) versus 1583 (344) mg/L; *P* = 0.951). There was also no difference in HDL_2_ and HDL_3_ apo AI in the groups with <8.34% or ≥8.34% HbA1c, comparison 2 (HDL_2_, 141 (56) versus 150 (48) mg/L; *P* = 0.508: HDL_3_, 1575 (356) versus 1591 (338) mg/L; *P* = 0.869).

### 3.3. hsCRP in Serum and SAA in Serum, HDL_2_, and HDL_3_

*Serum Analyses*. Serum-hsCRP did not differ between the groups in comparison 1 or 2 (*P* = 0.162 and *P* = 0.355, resp., Table 2). Although serum-SAA appeared higher in the overall T1DM group from comparison 1, this only approached significance compared to the control group (*P* = 0.088). However, in comparison 2, serum-SAA was statistically higher in the T1DM group with HbA1c ≥ 8.34%, compared to the T1DM group HbA1c < 8.34% (*P* = 0.031).

*HDL Analyses*. Compared to the control group, the protein standardised and nonstandardised SAA in HDL_2_ and HDL_3_ was statistically higher in the overall T1DM group from comparison 1 (*P* < 0.05, for both analyses). Comparison 2 illustrated that protein and nonprotein standardised HDL_2_-SAA, although appeared higher in the T1DM group with HbA1c ≥ 8.34%, it was not statistically different compared to the T1DM group with HbA1c < 8.34% (*P* = 0.096 and 0.085, resp.). However, both protein and nonprotein standardised HDL_3_-SAA was statistically higher in the T1DM group with HbA1c ≥ 8.34%, compared to the T1DM group with HbA1c < 8.34% (*P* = 0.028 and 0.019, resp.).

### 3.4. Relationship between HbA1c and SAA

**Figure 1:**
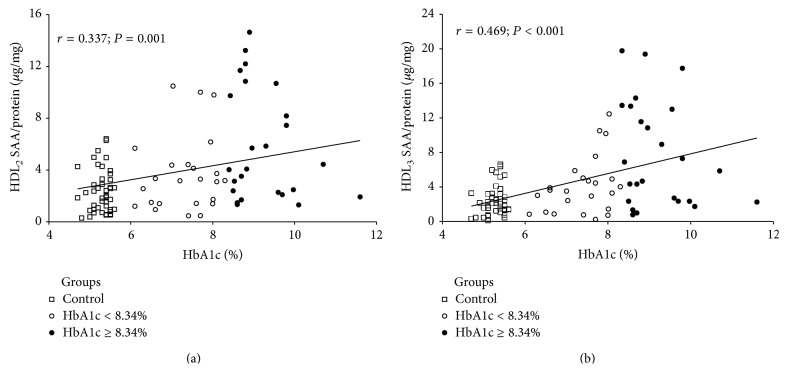
Relationship between HbA1c and SAA in HDL_2_ (a) and HDL_3_ (b).

The relationship between HbA1c and SAA was further examined by linear regression analysis. This revealed a positive relationship between increased HbA1c and both HDL_2_-SAA and HDL_3_-SAA (Figures 1(a) and 1(b), resp.; *P* < 0.001 for both analyses), where every 1% unit increase in HbA1c was associated with an estimated 20% (CI; 8–33%) increase in HDL_2_-SAA and an estimated 23% (CI; 10–37%) increase in HDL_3_-SAA. This relationship was further examined when we excluded the control group from this analysis. Here the positive relationship continued, although this was only significant for HDL_3_ (HDL_2_; *r* = 0.306, *P* = 0.098: HDL_3_; *r* = 0.452, *P* = 0.027). No relationship was found between HbA1c and serum-SAA (*r* = 0.187; *P* = 0.110). BMI, age, and gender did not impact on our model, meaning that they were not included in our regression analyses.

### 3.5. Correlations between SAA and hsCRP with Subject Characteristics

There was a strong positive correlation between serum-SAA and SAA associated with HDL_2_ and HDL_3_ (*P* ≤ 0.001 for both correlations, Table 3). In addition, the presence of T1DM was positively correlated with serum-SAA, HDL_2_-SAA, and HDL_3_-SAA, although this was only significant for the HDL subfractions (*P* = 0.093, 0.014 and 0.011, resp.). BMI was positively correlated with serum-SAA and HDL_3_-SAA (*P* = 0.002 for both correlations). Fasting plasma glucose, age, and gender were not correlated with serum-SAA, HDL_2_-SAA, or HDL_3_-SAA (*P* > 0.05 for all correlations). hsCRP was moderately correlated with serum-SAA (*P* = 0.038, Table 3) and negatively correlated with age (*P* = 0.007). HsCRP did not correlate with the presence of T1DM, glucose, BMI, or gender (*P* > 0.05 for all correlations, Table 3) or with HbA1c (*r* = 0.212; *P* = 0.075).

## 4. Discussion

This is the first reported study to examine SAA in HDL in subjects with T1DM, illustrating that SAA was increased in the main subtypes of HDL, HDL_2_, and HDL_3_. The antiatherogenic properties of HDL particles include their pivotal role in reverse cholesterol transport [15], as well as antioxidant [16] and anti-inflammatory effects [17]. However, in the presence of SAA-related inflammation, these antiatherogenic properties are impaired [18–20]. Following release into the circulation, SAA associates with HDL, particularly HDL_3_ [21], which can augment atherogenesis, as SAA enhances the binding of HDL to the arterial wall [22]. Furthermore, dysfunctional HDL displays reduced reverse cholesterol transport and antioxidant capabilities [23]. In the current study, SAA was increased by 53% in HDL_2_ and 58% in HDL_3_ in subjects with T1DM. This SAA had been standardised to total protein, which was 15% higher in HDL_2_ and 14% lower in HDL_3_, compared to the control subjects. These differences were small compared to differences in SAA and, therefore, were unlikely to have significantly influenced the HDL-SAA results. However, to ensure this was not the case we also included the nonprotein standardised SAA results, where the difference in HDL-SAA between the groups was maintained.

To examine the relationship between glycaemic control and HDL-SAA levels, we separated the overall T1DM group according to mean HbA1c (< or ≥8.34%), where the differences identified in comparison 1 was, in the main, driven by higher HDL-SAA levels in the T1DM subjects with poor glycaemic control (HbA1c ≥ 8.34%; comparison 2). Although prolonged poor glycaemic control is associated with chronic inflammation [24], the mechanisms underlying the association between increased SAA and T1DM are not clear. However, there is evidence that SAA promotes insulin resistance [25] and that intensive insulin therapy can reduce SAA levels [4], whilst the insulin-sensitising and antioxidant drug troglitazone is reported to lower SAA in T2DM subjects [26]. In support of the concept that glycaemic control and inflammation are linked, the current study further illustrated that glycaemic control was closely associated with increased SAA-related inflammation, where for every 1% unit increase in HbA1c there was a concomitant increase in HDL_2_ and HDL_3_-associated SAA of 20% and 23%, respectively. However, and contrary to this, Heliövaara et al. [6] reported that serum-SAA did not respond to glycaemic intervention to lower HbA1c in T1DM subjects, although we suggest that this may not have been the case had they measured HDL-SAA. Additionally, this group [6] only reported a 0.8% decrease in HbA1c following intervention, which may have been insufficient to mediate a statistical effect. Finally, their small subject population (24 subjects with T1DM) may have minimised their power to detect a change in serum-SAA, especially as serum-SAA is reported to display a large standard deviation (as discussed in detail below).

Overall, and to the author's knowledge, this is the first study to demonstrate an association between glycaemic control and levels of HDL-SAA in subjects with T1DM, and we suggest that a possible mechanism may be a result of an “underinsulinised” liver in poorly controlled T1DM subjects.

With regard to our serum-SAA results, although serum-SAA was higher in the poorly controlled T1DM subjects (HbA1c ≥ 8.34%) in comparison 2 (*P* = 0.031), between-group differences were less clear-cut between the overall T1DM subjects and the control subjects from comparison 1 (*P* = 0.088). We can suggest several reasons for this anomaly; firstly, the T1DM subjects with better glycaemic control (HbA1c < 8.34%; comparison 2) may have impacted on the T1DM result in comparison 1. Secondly, as serum-SAA displays a large variation, as illustrated by our wide spread of interquartile ranges reported in Table 2, this would minimise our statistical power. In fact, a large standard deviation was suggested to be responsible for the nonsignificantly higher levels of serum-SAA reported in 24 T1DM subjects, compared to 16 control subjects [6]. Further credence to this concept may be provided by Zhi et al. [5], where they reported statistically higher serum-SAA levels in a very large T1DM cohort (1139 T1DM subjects versus 848 control subjects). Furthermore, although Basu et al. [27] reported that serum-SAA was not different in a T1DM/control group comparison with similar subject numbers to our current study (38 T1DM subjects versus 41 control subjects), we suggest that this may be due to their control group being under greater inflammatory stress, as both serum-SAA and hsCRP were higher in their control group.

Overall, we suggest that the apparently lower sensitivity of serum-SAA, compared to the HDL subfractions, possibly reflects differences between acute and chronic inflammation. As described under Introduction, serum-SAA is indicative of both acute and chronic inflammation [7] and, therefore, more subject to daily fluctuations, which may impact greatly when subject numbers are small. In contrast, since HDL has a half-life of approximately 4 days [9], SAA associated with this lipoprotein is predicted to be more stable and, therefore, a more useful marker of chronic low grade inflammation in both small and large cohorts.

## 5. Conclusions

In summary, this cross-sectional study has highlighted HDL-SAA as a sensitive biomarker to detect increased inflammation in subjects with T1DM and provides a potential mechanistic explanation for accelerated atherosclerosis of this condition. Longitudinal studies are required to explore whether SAA changes in response to improved glycaemic control in T1DM.

*Study Limitations*. There are several limitations to this study. Firstly, we must address the fact that several of our T1DM patients were on statin therapy (10%), which is known to lower SAA levels, especially under conditions of heightened inflammation [28]. However, this was also the group in which SAA was increased, suggesting that SAA may have been even higher had none of the T1DM subjects been on this therapy. Secondly, several of our T1DM subjects were taking ACE inhibitors, which are reported to increase SAA [29]. However, although this may appear, in part, to have driven the difference between the T1DM and control cohorts, this could not be the case as ACE inhibitor use was similar in the two T1DM cohorts, while SAA levels were different. This would suggest that it was unlikely that the ACE inhibitors had influenced SAA levels in this study. Thirdly, our lack of an ability to detect statistically significant differences in serum-SAA between our overall T1DM group and the control group may be due to our relatively small subject numbers and the fact that SAA is known to display a large variation between subjects. Fourthly, we did not examine SAA associated with VLDL and LDL, which has recently been shown to be increased in subjects with documented atherosclerosis [30]. Fifthly, due to our small subject numbers meant that it was not possible to correlate SAA with established CVD risk factors, such as intimal media thickness. Sixthly, we also acknowledge that it is desirable for HbA1c to be ≤7% to reduce the vascular complications of diabetes and to indicate a better-controlled T1DM cohort [31]. However, in the current study only 5 of our 50 T1DM patients had an HbA1c of ≤7%, meaning that it was not possible to carry out such a sub analysis. Finally, we must address the differing HDL-protein concentrations identified between the control and T1DM groups, which we suggest may be related to changes in HDL-associated proteins, relative to T1DM status. In the case of HDL_2_, this may be related, in part, to the increased SAA protein identified in the T1DM subjects, and, in the case of HDL_3_, this may be related to changes in the concentration of other HDL-associated proteins, such as paraoxonase-1, which readily associates with HDL_3_ [32] and is influenced by inflammation [33].

## Figures and Tables

**Table 1:** Subject characteristics for comparison 1 and comparison 2.

Characteristic	Comparison 1			Comparison 2		
	Control group (*n* = 49)	T1DM group (*n* = 50)	*t*-test	T1DM group HbA1c <8.34% (*n* = 24)	T1DM group HbA1c ≥8.34% (*n* = 26)	*t*-test
BMI (kg/m^2^)	26.4 (3.5)	26.0 (3.9)	0.686	25.0 (3.5)	27.0 (4.1)	0.062
Age (years)	39.4 (10.1)	35.8 (8.3)	0.095	36.8 (9.1)	35.0 (7.5)	0.451
Gender (male : female)	17 : 32	18 : 32	0.836	9 : 15	9 : 17	0.836
Fasting glucose (mmol/L)	5.05 (4.60, 5.30)	9.89 (6.30, 14.60)	≤0.001	9.05 (5.20, 12.80)	10.63 (6.58, 14.85)	0.279
HbA1C (%)	5.3 (5.1, 5.5)	8.3 (7.4, 8.8)	≤0.001	7.4 (6.8, 8.0)	9.2 (8.6, 9.7)	≤0.001
HbA1c (mmol/mol)	34 (32, 37)	68 (57, 72)	≤0.001	57 (51, 64)	76 (70, 83)	≤0.001
Total cholesterol (mmol/L)	4.92 (0.86)	4.60 (0.84)	0.065	4.36 (0.83)	4.83 (0.80)	0.048
Triglycerides (mmol/L)	1.31 (0.77)	1.04 (0.39)	0.050	0.98 (0.34)	1.09 (0.44)	0.330
HDL cholesterol (mmol/L)	1.41 (0.34)	1.50 (0.41)	0.204	1.43 (0.52)	1.57 (0.28)	0.231
LDL cholesterol (mmol/L)	2.99 (0.84)	2.62 (0.68)	0.032	2.48 (0.57)	2.75 (0.76)	0.157
Statin therapy (number)	0	5	NA	1	4	NA
ACE Inhibitors (number)	0	5	NA	2	3	NA

Results expressed as mean (SD) or when data was not normally distributed as geometric mean (interquartile range).

**Table 2:** Serum hsCRP and Serum, HDL_2_ and HDL_3_ SAA concentration.

Analyate	Comparison 1			Comparison 2		
	Control group (*n* = 49)	T1DM group (*n* = 50)	*t*-test	T1DM group HbA1c <8.34% (*n* = 24)	T1DM group HbA1c ≥8.34% (*n* = 26)	*t*-test
Serum-hsCRP (mg/L)	1.97 (1.00, 2.38)	2.89 (1.00, 4.40)	0.162	2.56 (1.00, 2.30)	3.24 (1.30, 4.93)	0.355
Serum-SAA (*µ*g/L)	16241 (7275, 17645)	23837 (8133, 40737)	0.088	15885 (8060, 18945)	30706 (7984, 47986)	0.031
Protein-standardised SAA						
HDL_2_ (*µ*g/mg protein)	3.00 (0.97, 4.12)	5.63 (1.65, 7.63)	0.003	4.97 (1.41, 4.42)	6.23 (2.06, 9.94)	0.096
HDL_3_ (*µ*g/mg protein)	3.48 (1.37, 3.55)	5.95 (2.31, 9.57)	0.005	4.50 (1.18, 5.01)	7.34 (2.35, 12.29)	0.028
Nonprotein standardised SAA						
HDL_2_ (*µ*g/L)	690 (242, 951)	1674 (379, 1990)	0.004	1260 (357, 1408)	2055 (541, 2589)	0.085
HDL_3_ (*µ*g/L)	8951 (3326, 8966)	15972 (4625, 23331)	0.005	10362 (3883, 13315)	21151 (6846, 33534)	0.019

Results expressed as mean (SD) or when data was not normally distributed as geometric mean (interquartile range).

**Table 3:** Correlations between SAA and subject characteristics.

	Serum-SAA *r* =; *P* =	HDL_2_-SAA *r* =; *P* =	HDL_3_-SAA *r* =; *P* =	hsCRP *r* =; *P* =
Serum-SAA	—	**0.779**; ≤**0.001**	**0.888**; ≤**0.001**	**0.234**; **0.038**
T1DM status	0.191; 0.093	**0.246**; **0.014**	**0.256**; **0.011**	0.206; 0.062
BMI	**0.349**; **0.002**	0.044; 0.663	**0.302**; **0.002**	0.196; 0.076
Fasting plasma glucose	0.063; 0.730	0.134; 0.457	0.113; 0.539	−0.124; 0.267
Age	0.070; 0.541	−0.056; 0.585	−0.011; 0.917	**−0.294**; **0.007**
Gender	0.197; 0.082	0.139; 0.169	0.155; 0.129	0.077; 0.491

T1DM status: control = 0; type 1 = 1.

## Abbreviations

CVD: Cardiovascular disease
HbA1c: Glycated haemoglobin
HDL: High-density lipoprotein
hsCRP: High-sensitivity C-reactive protein
SAA: Serum amyloid A
T1DM: Type 1 diabetes mellitus.
